# Moderate PM_10_ exposure increases prostate cancer: a longitudinal nationwide cohort study (2010–2020)

**DOI:** 10.3389/fpubh.2024.1490458

**Published:** 2025-01-10

**Authors:** Mi Jung Rho, Yong Hyun Park, Jihwan Park

**Affiliations:** ^1^College of Health Science, Dankook University, Cheonan-si, Chungcheongnam-do, Republic of Korea; ^2^Department of Urology, Seoul St. Mary's Hospital, College of Medicine, The Catholic University of Korea, Seoul, Republic of Korea; ^3^College of Liberal Arts, Dankook University, Cheonan-si, Chungcheongnam-do, Republic of Korea

**Keywords:** particulate matter exposure, prostate cancer, PM_10_, the National Health Insurance Sharing Service, national cohort study

## Abstract

**Introduction:**

Fine dust exposure has been reported to affect patients with prostate cancer, making it crucial to understand how environmental pollutants impact health. This study aimed to determine the risk of prostate cancer in South Korea associated with moderate levels of fine dust (PM_10_) exposure.

**Methods:**

We analyzed data from 20,430 individuals in the National Health Insurance Sharing Service database from 2010 to 2020, comparing a new prostate cancer group (*n* = 4,071, 19.9%) with a non-prostate cancer group (*n* = 16,359, 80.1%). Using PM_10_ data from Air Korea's annual average air quality database, we conducted logistic regression analysis to assess the risk of prostate cancer.

**Results:**

Our findings indicate that even moderate PM_10_ exposure is a risk factor for developing prostate cancer. Additionally, even at low levels of PM_2.5_, moderate PM_10_ exposure significantly impacts prostate cancer development, with lifestyle ha bits potentially lowering this risk.

**Discussion:**

These results underscore the need for stricter environmental standards for PM_10_ and proactive policies to reduce public health and long-term social costs. Public awareness, including mask use and air quality management, is essential.

## 1 Introduction

Environmental pollutants, particularly particulate matter (PM) air pollution, have been increasingly recognized for their significant impact on human health. Numerous studies have established a strong correlation between environmental pollution and various types of cancer, including lung, heart, brain, and respiratory diseases (1). Among these pollutants, fine dust, specifically PM_10_, has been implicated in a range of diseases such as chronic obstructive pulmonary disease (COPD), asthma, and lung cancer (2–6). Fine dust penetrates deep into the lungs, causing inflammation and increasing the risk of lung cancer. Additionally, emerging research suggests that fine dust exposure may also contribute to the development of urological cancers, including kidney cancer, urothelial cell carcinoma, and prostate cancer (PCa) (7, 8). Some study have also found that renal function and chronic urologic diseases are associated with PM_10_ (9, 10). There are also paper on the relationship between industrial pollution, including PM_10_, and the risk of PCa (11). However, research on PCa and PM_10_ is still needed, and diverse well-designed studies on PCa are needed. In addition, in Korea, there is a public DB on air quality (the Air Korea's annual average air quality database), so research using it is possible.

While various environmental standards for fine dust exist globally, these standards vary by country, reflecting local conditions. In Korea, the environmental standards for fine dust have adopted the second stage of the World Health Organization's (WHO) 2005 interim targets for air quality (12). According to the 2021 WHO recommendation, the PM_10_ standard levels is 15 μg/m^2^ for the annual average and 45 μg/m^2^ for the 24-h average (12). However, in Korea, the PM_10_ standard levels is 50 μg/m^2^ for the annual average and 100 μg/m^2^ for the 24-h average (13). Despite efforts to reduce fine dust levels through various policies, Korea's standard levels concentration remains high compared to that in developed countries.

PCa is a significant health concern, particularly in Korea, where it ranks third among cancers in men and sixth in overall cancer incidence as of 2021 (14). In Western countries such as Europe and the United States, it has been the number one cancer in men for a long time (15). PCa is rapidly increasing along with the increase in the aging population. Its importance is increasing in Korea, which is entering a super-aging society. PCa is a cancer with a good prognosis when detected early, with a high survival rate of more than 5 years. However, the social costs caused by PCa remain enormous.

Given the rising incidence of PCa and the ongoing concerns about environmental pollution, it is crucial to understand the potential role of fine dust exposure in the development of this cancer. Therefore, this study aimed to investigate the risk of PCa due to moderate level fine dust (31–80 μg/m^2^) concentration in Korea (13).

## 2 Methods

### 2.1 Hypothesis

Previous research has established that PM_10_ is associated with various diseases, including respiratory illnesses, lung cancer, heart disease, brain disorders, chronic obstructive pulmonary disease, asthma, COPD exacerbation, esophageal cancer, and corpus uteri cancer (6, 16). Particulrly, PM_10_ is a risk factor for the development of urological cancers, including kidney cancer, PCa, and urothelial cell carcinoma (7, 17). This study hypothesized that even moderate exposure to PM_10_ could influence the risk of developing PCa. The specific hypothesis was as follows.

H1: Moderate level PM_10_ exposure has relationship with PCa.

### 2.2 Data source

We used customized health information data from the National Health Insurance Sharing Service (NHISS). In Korea, the NHISS discloses “customized health information data” to researchers (18). Customized health information refers to data provided by processing citizens' health information collected, held, and managed by the NHISS into customized data that can be used for policy and academic research purposes. The NHISS provides researchers with a sample cohort database (DB), health examination cohort DB and older adult cohort DB. The entire data period provided by the NHISS was from 2002 to 2020 (as of 2023, when this study was first conducted). This study used data from 2010 to 2020.

### 2.3 Study population

**Figure 1:**
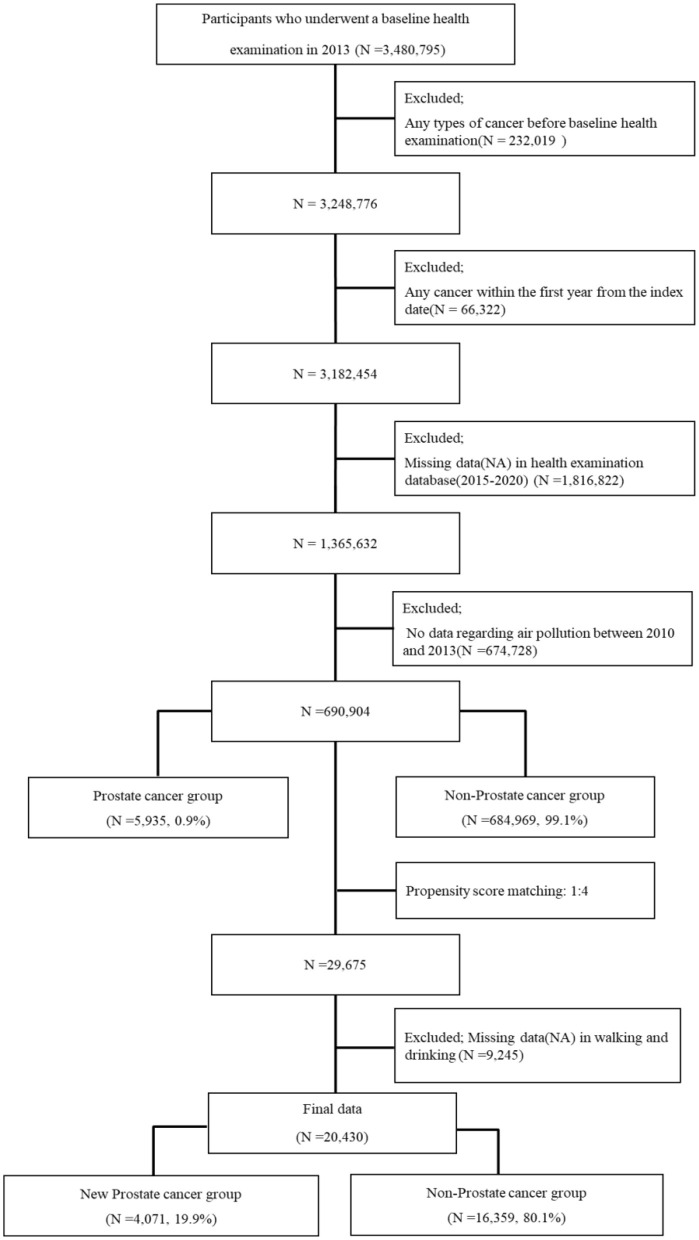
Flowchart of participant inclusion.

Participants were selected from the NHISS database, including individuals who underwent a baseline health examination in 2013 (*n* = 3,480,795). Exclusions were made as follows: participants diagnosed with cancer before the baseline examination (*N* = 232,019), those diagnosed with cancer in the 1st year (*n* = 66,322), participants with missing health examination data between 2015 and 2020 (*n* = 1,816,822), and those with no data on air pollution between 2010 and 2013 (*n* = 674,728). The final cohort consisted of 690,904 participants, with 5,935 cases (0.9%) in the PCa group and 684,969 (99.1%) in the non-PCa group. Propensity score matching (PSM) was applied to minimize selection bias, resulting in 29,674 matched participants. After excluding cases with missing data on weekly walking and drinking habits, 20,430 participants were included in the final analysis, divided into PCa (*n* = 4,071, 19.9%) and non-PCa groups (*n* = 16,359, 80.1%; Figure 1).

**Figure 2:**
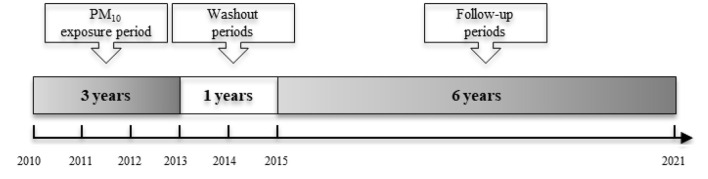
Participants' timeline.

The period of PM_10_ exposure covered 3 years, from 2010 to 2012 (Figure 2). A 1-year washout period followed. The follow-up period for the target data spanned 6 years, from 2015 to 2020.

### 2.4 Definition of prostate cancer

PCa was classified under ICD-10 code C61. In this study, PCa cases were identified based on this classification, with a total of 4,071 patients diagnosed with PCa.

### 2.5 Particulate matter exposure (PM_10_)

PM_10_ exposure data were obtained from the Air Korea's annual average air quality database, which monitors air quality across various administrative districts in Korea. The Air Korea's annual average air quality database is categorized by area code. It was used by matching the area code of the subject in the NHISS database. PM_10_ refers to particulate matter with a diameter of 10 μm or less (19). In Korea, fine dust forecast grades are divided into four levels: good (0–30 μg/m^3^), moderate (31–80 μg/m^3^), bad (81–150 μg/m^3^), and very bad (over 151 μg/m^3^) (13).

### 2.6 Covariate assessment

Eight covariates were assessed: weekly walking frequency, alcohol consumption, smoking status, hypertension, diabetes mellitus, hyperlipidemia, body mass index (BMI), and PM_2.5_ exposure. These variables were categorized as follows: Walking per week: (1) no walking, (2) 1–2 times per week, (3) 3–4 times per week, and (4) 5–7 times per week. Drinking: (1) no drinking, (2) ~2–3 times a month, (3) ~1–2 times a week, (4) 3–4 times a week, and (5) almost daily. Smoking status: (1) non-smokers, (2) ex-smokers, and (3) smokers. Hypertension was classified into three types based on systolic blood pressure and diastolic blood pressure values: (1) normal group [systolic blood pressure (sbp): <120 and diastolic blood pressure (dbp): <80], (2) normal border group (sbp: 120–139 or dbp: 80–89), and (3) group suspected of hypertension (sbp: ≥140 or dbp: ≥90) (20). Diabetes mellitus was divided into three types based on fasting blood sugar: (1) normal (<100 mg/dL), (2) prediabetes (100–125 mg/dL), and (3) diabetes (≥126 mg/dL) (21). Hyperlipidemia was divided into three types based on total cholesterol: (1) low (under 200), (2) middle (200–239), and (3) high (≥240) (22). BMI was divided into three types: (1) normal weights (18.5–24.9), (2) normal weight-border (<18.5 or 25–29.9), and (3) obesity (BMI of 30 or greater) (23). PM_2.5_ exposure was classified into two groups based on 25 μg/m^3^: (1) low exposure group (Under 25 μg/m^3^) and (2) high exposure group (Under 25 μg/m^3^). The average value of PM_2.5_ was 25.48 μg/m^3^ and the median value was 24.79 μg/m^3^. Therefore, the groups were divided based on the value of 25 μg/m^3^.

### 2.7 Statistical analysis

Statistical analysis was conducted using R software for data preprocessing and Propensity Score Matching (PSM), and SPSS for basic statistical analysis, chi-square tests, and multivariate logistic regression. The chi-square test was used to compare demographic characteristics by PM_10_ exposure levels, and logistic regression analysis was used to assess the risk of PCa associated with PM_10_ exposure. All statistical tests were two-tailed, and the significance level was set at *P*-values < 0.05. The analyses were performed using R version 4.0.4 (2020-10-10) and IBM Statistical Package for the Social Sciences (SPSS) Statistics (version 25.0; SPSS Inc.).

## 3 Results

### 3.1 Participant characteristics

The study included 20,430 participants, with 67.4% aged under 65 years and 32.6% over 65 years (Table 1). The average age was 60.76 years, and the median age was 61 years. Regarding physical activity, 22.3% of participants did not engage in any walking, while 32.5% walked more than 5 times per week. Overall, 77.7% of participants walked at least once per week. Additionally, 30.8% of participants consumed alcohol two to three times per month, and 12.4% abstained from drinking entirely. The analysis also revealed that 39.9% of participants were ex-smokers, and 29.1% were non-smokers. Hypertension was suspected in 19.5% of the participants, which aligns with the global variation in hypertension prevalence, reported to be 18% in America and 27% in Africa (20). The majority (51.4%) had a total cholesterol level below 200 mg/dL, and 58.9% had fasting blood sugar levels under 100 mg/dL. A total of 63.4 63.4% of participants were classified within the normal BMI range (18.5 to 24.9). A total of 80.1% belonged to the No PCa group and 19.9% belonged to the PCa group.

**Table 1:** Characteristics of the participants.

**Variables**		**Frequency**	**Percent**
Age	<65 years	13,768	67.4
	≥65 years	6,662	32.6
Walking per week	No walking	4,561	22.3
	1–2 times a week	4,377	21.4
	3–4 times a week	4,861	23.8
	5–7 times a week	6,631	32.5
Drinking	No-drinking	2,532	12.4
	About 2–3 times a month	6,297	30.8
	About 1–2 times a week	5,004	24.5
	3–4 times a week	4,452	21.8
	Almost everyday	2,145	10.5
Smoking	Non-smoker	5,936	29.1
	Ex-smoker	8,153	39.9
	Smoker	6,341	31.0
Hypertension (systolic blood pressure, diastolic blood pressure)	Normal group (^*^sbp: < 120 and ^*^dbp: < 80)	4,991	24.4
	Normal border group (sbp: 120–139 or dbp: 80–89)	11,450	56.0
	Group suspected of hypertension (sbp: ≥140 or dbp: ≥90)	3,989	19.5
Diabetes mellitus (fasting blood sugar, mg/dL)	Normal (<100)	10,500	51.4
	Prediabetes (100–125)	7,452	36.5
	Diabetes (≥126)	2,478	12.1
Hyperlipidemia (total cholesterol, mg/dL)	Under 200	12,043	58.9
	200–239	6,339	31.0
	≥240	2,048	10.0
^*^BMI (kg/m^2^)	Normal weight (18.5–24.9)	12,948	63.4
	Normal weight-border (<18.5 or 25–29.9)	7,036	34.4
	Obesity (BMI of 30 or greater)	446	2.2
PM_2.5_ exposure group	Under 25 μg/m^3^	10,673	52.2
	Over 25 μg/m^3^	9,757	47.8
PM_10_ exposure group	Low exposure group (Under 47 μg/m^3^)	9,920	48.6
	High exposure group (Over 47 μg/m^3^)	10,510	51.4
Group	No PCa group	16,359	80.1
	PCa group	4,071	19.9
Total		20,430	100

^*^sbp, systolic blood pressure; dbp, diastolic blood pressure; BMI, body mass index.

### 3.2 PM_10_ exposure and baseline statistics

**Figure 3:**
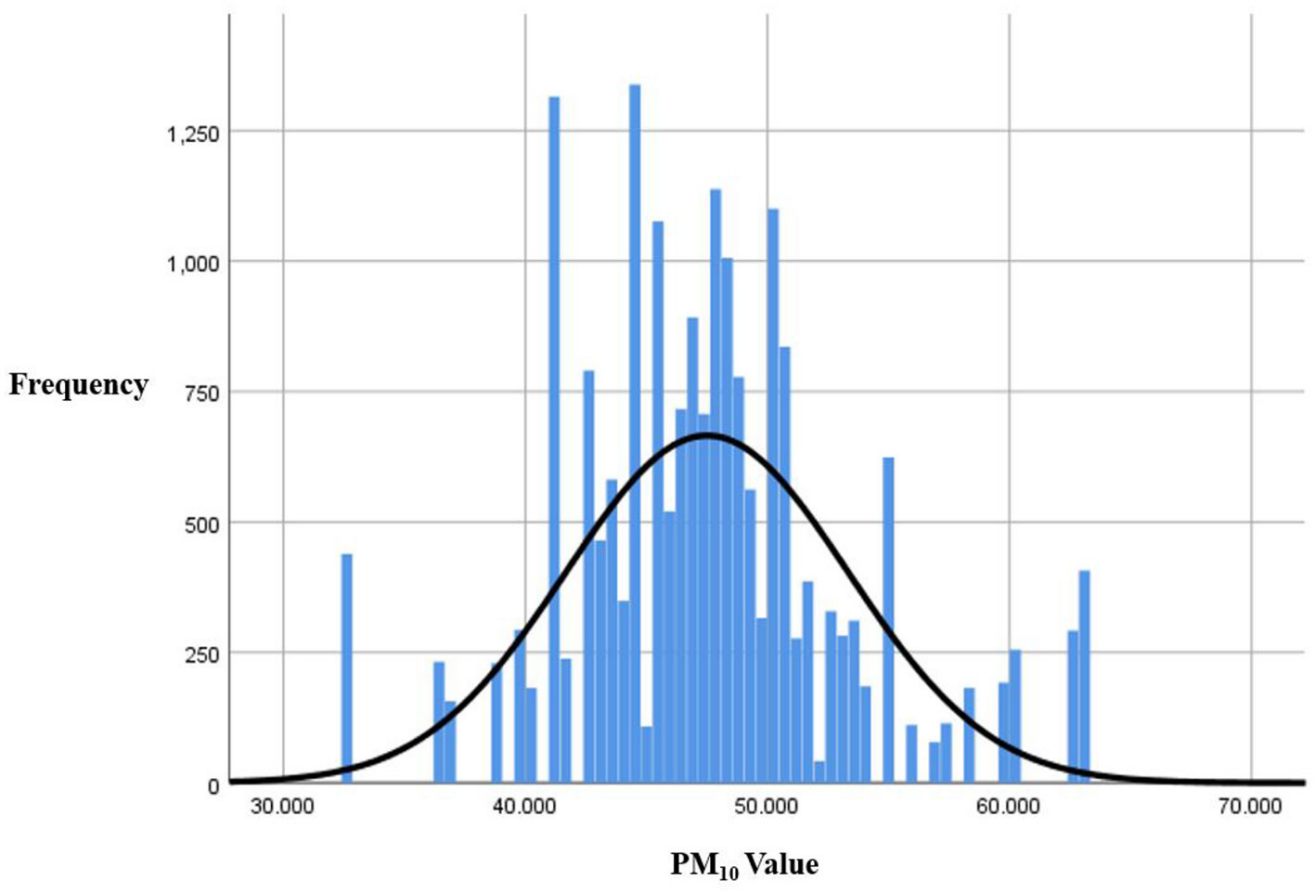
Particulate matter distribution (PM_10_).

The average PM_10_ exposure level was 47.48 μg/m^3^, with a median of 47.49 μg/m^3^. Participants were categorized into low and high PM_10_ exposure groups based on a threshold of 47 μg/m^3^. The minimum exposure level recorded was 32.44 μg/m^3^, and the maximum was 62.92 μg/m^3^. The skewness and kurtosis values were 0.345 and 0.851, respectively, indicating a relatively normal distribution. According to Korea's air quality classification, these values correspond to an “average” level of PM_10_ exposure (31–80 μg/m^3^) (Figure 3).

Table 2 shows the demographics according to PM_10_ exposure. The PCa and non-PCa groups were divided into two groups according to PM_10_ exposure. In the non-PCa group, significant differences were observed in weekly walking (*P* = 0.002), drinking (*P* < 0.001), smoking (*P* = 0.009), BMI (*P* = 0.034), and PM_2.5_ exposure (*P* < 0.001). In the PCa group, there were significant differences in walking per week (*P* = 0.002) and PM_2.5_ exposure (*P* < 0.001) between low PM_10_ exposure and high PM_10_ exposure.

**Table 2:** Demographics according to the PM_10_ exposure.

**Variables**		**Non-PCa group (*****N*** = **16,359)**			**PCa group (*****N*** = **4,071)**		
		**Low PM**_10_ **exposure**	**High PM**_10_ **exposure**	***p*** **-value**	**Low PM**_10_ **exposure**	**High PM**_10_ **exposure**	***p*** **-value**
No. of patients		8,008 (49%)	8,351 (51%)	16,359 (100%)	1,912 (47%)	2,159 (53%)	4,071 (100%)
Age	<65 years	5,413 (68%)	5,666 (68%)	0.729	1,237 (65%)	1,452 (67%)	0.086
	≥65 years	2,595 (32%)	2,685 (32%)		675 (35%)	707 (33%)	
Walking per week	No walking	1,698 (21%)	1,919 (23%)	0.002^**^	397 (21%)	547 (25%)	0.002^**^
	1–2 times a week	1,663 (21%)	1,831 (22%)		408 (21%)	475 (22%)	
	3–4 times a week	1,962 (25%)	1,915 (23%)		483 (25%)	501 (23%)	
	5–7 times a week	2,685 (34%)	2,686 (32%)		624 (33%)	636 (29%)	
Drinking	No-drinking	867 (11%)	1,107 (13%)	0.000^***^	243 (13%)	315 (15%)	0.137
	About 2–3 times a month	2,500 (31%)	2,481 (30%)		651 (34%)	665 (31%)	
	About 1–2 times a week	1,962 (25%)	2,048 (25%)		468 (24%)	526 (24%)	
	3–4 times a week	1,800 (22%)	1,831 (22%)		371 (19%)	450 (21%)	
	Almost everyday	879 (11%)	884 (11%)		179 (9%)	203 (9%)	
Smoking	Non-smoker	2,351 (29%)	2,293 (27%)	0.009^**^	633 (33%)	659 (31%)	0.124
	Ex-smoker	3,126 (39%)	3,267 (39%)		797 (42%)	963 (45%)	
	Smoker	2,531 (32%)	2,791 (33%)		482 (25%)	537 (25%)	
Hypertension (systolic blood pressure, diastolic blood pressure)	Normal group (sbp: <120 and dbp: <80)	1,896 (24%)	2,041 (24%)	0.399	508 (27%)	546 (25%)	0.234
	Normal border group (sbp: 120–139 or dbp: 80–89)	4,520 (56%)	4,630 (55%)		1,087 (57%)	1,213 (56%)	
	Group suspected of hypertension (sbp: ≥140 or dbp: ≥90)	1,592 (20%)	1,680 (20%)		317 (17%)	400 (19%)	
Diabetes mellitus (fasting blood sugar, mg/dL)	Normal (<100)	4,025 (50%)	4,299 (51%)	0.286	1,012 (53%)	1,164 (54%)	0.700
	Prediabetes (100–125)	2,970 (37%)	3,010 (36%)		694 (36%)	778 (36%)	
	Diabetes (≥126)	1,013 (13%)	1,042 (12%)		206 (11%)	217 (10%)	
Hyperlipidemia (total cholesterol, mg/dL)	Under 200	4,693 (59%)	4,975 (60%)	0.252	1,090 (57%)	1,285 (60%)	0.182
	200–239	2,521 (31%)	2,529 (30%)		617 (32%)	672 (31%)	
	≥240	794 (10%)	847 (10%)		205 (11%)	202 (9%)	
BMI (kg/m^2^)	Normal weight (18.5–24.9)	5,079 (63%)	5,365 (64%)	0.034^*^	1,172 (61%)	1,332 (62%)	0.415
	Normal weight-border (<18.5 or 25–29.9)	2,727 (34%)	2,824 (34%)		707 (37%)	778 (36%)	
	Obesity (BMI of 30 or greater)	202 (3%)	162 (2%)		33 (2%)	49 (2%)	
PM_2.5_ exposure group	Low exposure group (Under 25 μg/m^3^)	5,367 (67%)	3,115 (37%)	0.000^***^	1,264 (66%)	927 (43%)	0.000^***^
	High exposure group (Under 25 μg/m^3^)	2,641 (33%)	5,236 (63%)		648 (34%)	1,232 (57%)	

^*^P < 0.05; ^**^P < 0.01; ^***^P < 0.001.

### 3.3 Risk of prostate cancer by PM_10_ exposure

Table 3 presents the odds ratios (95% CI) for PCa according to moderate PM_10_ exposure, categorized into low (9,920 participants) and high (10,510 participants) exposure groups. Moderate PM_10_ exposure was identified as a significant predictor of PCa in all models: Model 1 (OR = 1.118), Model 2 (OR = 1.119), and Model 3 (OR = 1.121). These models were adjusted for various factors, including age, PM_2.5_, drinking habits, walking frequency, smoking status, hypertension, diabetes, hyperlipidemia, and BMI.

**Table 3:** Odds ratio of PCa according to the moderate PM_10_ exposure.

**PM_10_**	**Event**	**Odds ratio (95% CI)**		
		**Model 1**	**Model 2**	**Model 3**
Low exposure group (under 47 μg/m^3^)	9,920	1 (ref.)	1 (ref.)	1 (ref.)
High exposure group (under 47 μg/m^3^)	10,510	1.118 (95% CI: 1.041, 1.201)	1.119 (95% CI: 1.042, 1.203)	1.121 (95% CI: 1.043, 1.204)
*P* for trend		0.002^**^	0.002^**^	0.002^**^

Model 1 was adjusted for age, PM_2.5_. Model 2 was adjusted for age, PM_2.5_, alcohol consumption, walking, and smoking. Model 3 was adjusted for age, PM_2.5_, alcohol consumption, walking, smoking, Hypertension, Diabetes mellitus, Hyperlipidemia, and BMI.

** P < 0.01.

### 3.4 Risk of prostate cancer in clinically relevant subgroups

The subgroup results according to moderate exposure to PM_10_ are as follows: Not all the sub-variables were vulnerable to moderate PM_10_ exposure (Table 4). However, some subgroups were found to be vulnerable to even moderate exposure to PM_10_: under 65 years group (OR =1.121), under 25 μg/m^3^ group (OR = 1.264), no walking group (OR = 1.219), ex-smoker group (OR = 1.156), group suspected of hypertension (OR = 1.196), under 200 of hyperlipidemia (OR = 1.112), obesity group (BMI of 30 or greater) (OR = 1.851), and drinking group 3–4 times a week (OR = 1.192).

**Table 4:** Odds ratio of PCa in relevant subgroups.

**Factors**		**Low exposure group (under 47 μg/m^3^)**	**High exposure group (under 47 μg/m^3^)**	**Sig**.
		**Reference**	**Odds ratio (95% CI)**	
Age	**<65 years**	**1 (ref.)**	**1.121 (1.031–1.220)**	**0.008** ^**^
	≥65 years	1 (ref.)	1.012 (0.899–1.140)	0.840
PM_2.5_	**Under 25** **μg/m**^**3**^	**1 (ref.)**	**1.264 (1.148–1.390)**	**0.000** ^***^
	Over 25 μg/m^3^	1 (ref.)	0.959 (0.863–1.066)	0.438
Walking per week	**No walking**	**1 (ref.)**	**1.219 (1.055–1.409)**	**0.007** ^**^
	1–2 times a week	1 (ref.)	1.057 (0.912– 1.226)	0.460
	3–4 times a week	1 (ref.)	1.063 (0.924– 1.222)	0.394
	5–7 times a week	1 (ref.)	1.019 (0.901– 1.152)	0.765
Smoke	Non–smoker	1 (ref.)	1.067 (0.944–1.207)	0.300
	**Ex–smoker**	**1 (ref.)**	**1.156 (1.040– 1.285)**	**0.007** ^**^
	Smoker	1 (ref.)	1.010 (0.883–1.155)	0.881
Hypertension (systolic blood pressure, diastolic blood pressure)	Normal group (sbp: <120 and dbp: <80)	1 (ref.)	0.998 (0.871–1.144)	0.982
	Normal border group (sbp: 120–139 or dbp: 80–89)	1 (ref.)	1.089 (0.994–1.194)	0.067
	**Group suspected of hypertension (sbp:** **≥140 or dbp:** **≥90)**	**1 (ref.)**	**1.196 (1.016–1.407)**	**0.031** ^*^
Diabetes mellitus (fasting blood sugar, mg/dL)	Normal (<100)	1 (ref.)	1.077 (0.980–1.184)	0.125
	Prediabetes (100–125)	1 (ref.)	1.106 (0.987–1.240)	0.083
	Diabetes (≥126)	1 (ref.)	1.024 (0.831–1.263)	0.824
Hyperlipidemia (total cholesterol, mg/dL)	**Under 200**	**1 (ref.)**	**1.112 (1.016–1.217)**	**0.021** ^*^
	200–239	1 (ref.)	1.086 (0.961–1.227)	0.188
	≥240	1 (ref.)	0.924 (0.743–1.148)	0.474
BMI (kg/m^2^)	Normal weight (18.5–24.9)	1 (ref.)	1.076 (0.986–1.174)	0.101
	Normal weight–border (<18.5 or 25–29.9)	1 (ref.)	1.063 (0.948–1.192)	0.299
	**Obesity (BMI of 30 or greater)**	**1 (ref.)**	**1.851 (1.137–3.014)**	**0.013** ^*^
Drinking	No–drinking	1 (ref.)	1.015 (0.840–1.227)	0.876
	About 2–3 times a month	1 (ref.)	1.029 (0.912–1.162)	0.641
	About 1–2 times a week	1 (ref.)	1.077 (0.937–1.237)	0.297
	**3–4 times a week**	**1 (ref.)**	**1.192 (1.024–1.388)**	**0.023** ^*^
	Almost everyday	1 (ref.)	1.128 (0.904–1.407)	0.288

^*^P < 0.05.

^**^P < 0.01.

^***^P < 0.001.

The bold values are significant results.

## 4 Discussion

This study aimed to determine the risks associated with moderate levels of PM_10_ exposure for the development of PCa, based on the fine dust standards of the Korean Ministry of Environment (13). The findings reveal several important insights.

First, we observed that even moderate levels (31–80 μg/m^3^, Korea standard) (13) of PM_10_ exposure are significant risk factors for the development of PCa. According to the Korean air-quality classifications, a PM_10_ level of 47 μg/m^3^ is considered moderate level, a level at which masks are typically not worn by the public (13). However, our findings suggest that even at these moderate levels, PM_10_ exposure can influence the development of urological cancers such as PCa. This aligns with previous studies that have highlighted detrimental effects of PM_10_ on cancer risk (7, 24). There is little research on moderate level PM_10_, so in-depth discussion on it is limited. However, it is very meaningful that disease risks were predicted even for moderate level fine dust, even based on Korean standards.

In Korea, days with poor air quality have increased since 2012 due to an increase in PM, and various measures have been sought along with public concerns (9). Since 2015, air quality alerts have increased nationwide (25). Nevertheless, as described in the introduction, Korea PM_10_ standards are less stringent than those in other countries. The PM_10_ standard levels of 2021 WHO recommendation is 15 μg/m^3^ for the annual average and 45 μg/m^3^ for the 24-h average (12). The PM_10_ standard levels is 50 μg/m^3^ for the annual average and 100 μg/m^3^ for the 24-h average in Korea (13). This study emphasizes that Korea should further strengthen air quality standards for PM_10_.

In addition, it is critical to implement more detailed guidelines and actively promote the use of masks and air purification strategies when air quality deteriorates to hazardous levels. Prior research has demonstrated that education about the health risks associated with fine dust can influence public perceptions of susceptibility and severity (26). Our findings support the need for robust awareness campaigns to mitigate these risks.

Second, the study highlights the importance of individual physical characteristics in the relationship between PM_10_ exposure and PCa development. We found that the risk of PCa due PM_10_ exposure is higher in patients under 65 years of age, in those with suspected hypertension, in individuals with total cholesterol levels below 200 mg/dL, and in obese individuals. These findings contribute to the ongoing debate on the association between obesity, air pollution, and cancer risk (27). Previous research continues to explore the relationship between local environment factors and cancer incidence (28). Many studies have suggested that air pollution may negatively contribute to body weight status in adults (29–33), our results also indicate that in obesity, PM_10_ exposure is a significant risk factor for PCa. Thus, identifying high-risk groups based on major factors such as underlying health conditions is crucial, and targeted mid- to long-term support for these groups should be prioritized.

Third, lifestyle habits also play a crucial role in the relationship between PM_10_ exposure and PCa. Our study found that the risk of developing PCa due to PM_10_ exposure is elevated in individuals who do not engage in regular exercise, in ex-smokers, and in those who consume alcohol three to four times a week. These results are consistent with previous research indicating that lifestyle management can help mitigate the incidence of cancer related to air pollution (7, 28, 34, 35). In order to reduce the social cost of cancer, a comprehensive solution approach is needed that addresses environmental issues such as air pollution and actively corrects people's lifestyle habits.

Finally, we observed that the risk of developing PCa due to PM_10_ exposure was higher even in the presence of low PM_2.5_. This result was very interesting because even when PM_2.5_ is low, increased PM_10_ exposure increases the risk of developing PCa. Both PM_10_ and PM_2.5_ are air quality measurements, and although they are very important air quality measurement factors and global challenge issues (36–40), they should be viewed separately. Depending on the disease, PM_2.5_ and PM_10_ exposure often affect the disease at the same time (40). In fact, there are days when PM_2.5_ levels are low but PM_10_ levels are high. Although it is important to understand the impact of the integrated air quality index or multiple pollutants together in some cases (41), the fact that PM_10_ alone still increases the risk of disease is very significant.

Despite these significant findings, our study has several limitations. First, air pollution encompasses a range of pollutants, including PM_10_, NO_2_, and PM_2.5_ (42), and the incidence of various cancers has been consistently linked to PM_2.5_ exposure (43, 44). However, our study focused mainly on the relationship between PM_10_ exposure and PCa. Future research should consider a broader range of air pollutants to provide a more comprehensive understanding of their combined effects. Second, the study limited the period of PM_10_ exposure to 3 years (2010–2012). We used PM_10_ exposure data from Air Korea's annual average air quality database. The Air Korea's annual average air quality database has been providing air quality data since 2009, we identified appropriate data for the study and used air quality data from 2010 to 2012, which provided the longest follow-up period. Therefore, we used only 3 years of data to select data with as many area codes as possible that can be matched with NHISS customized health information data and with PM_10_ values that can be used in research. Additional research with longer exposure periods is needed to fully assess the long-term risks associated with fine dust exposure. Third, this study considered some lifestyle factors such as alcohol consumption, smoking, and physical activity. However, there may be other lifestyle factors, such as diet and occupational exposure, which could also influence the risk of prostate cancer. However, the customized health information data from NHISS does not provide additional lifestyle data (such as diet and occupational exposure), so it could not be utilized. Future research is needed to derive risk factors by including various lifestyles. Fourth, this study is based on Korea's environmental standards (13), and since environmental standards may differ from country to country, there may be limitations in the global applicability of the research results. Future research needs to be conducted based on global standards. Fifth, there is research that shows a relationship between air pollution and temperature (45). If we can secure data on temperature in the future, it would be desirable to analyze various environmental factors such as temperature in addition to air quality.

Despite these limitations, this study provides important insights into the relationship between moderate PM_10_ exposure and increased PCa risk. The findings also underscore the need for more stringent and detailed environmental standards for PM_10_. Reducing the disease risk from fine dust requires a mid- to long-term, comprehensive solution approach to identify high-risk groups and reduce social costs. In addition, public health should emphasize the importance of wearing masks and managing air quality, and managing lifestyle habits to protect the negative health effects of fine dust exposure.

## Data Availability

The national health insurance sharing service requires approval of institutional review board. Requests to access these datasets should be directed to NHISS bigdata service, 0075030@nhis.or.kr.
